# Supplementary material for: Structural basis for clearing of ribosome collisions by the RQT complex
Source: Nat Commun. 2023 Feb 17;14:921. doi: 10.1038/s41467-023-36230-8 (PMC9938168; doi:10.1038/s41467-023-36230-8)
Supplement: Supplementary file 3 — Description of Additional Supplementary Files [file 41467_2023_36230_MOESM3_ESM.pdf]

File name: Supplementary Movie 1

Description: Comparison of RQT-bound ribosomes in state C1 and C2.

The video shows a morph between molecular models of RQT-bound ribosomes in the two observed states. In state 1, the ribosome adopts a POST state with P/P-tRNA, and RQT is bound to the small ribosomal subunit on top of rRNA helix 16. The ribosome in state 2 contains a tRNA in unusual pe/E state and the 40S head is swiveled by approximately 20 degrees. This head conformation is similar to a translocation intermediate usually observed in the presence of eEF2, while the position of the 40S body remains comparable to a POST state ribosome. The head swivel is accompanied by a downwards movement of Slh1 on rRNA helix 16.

In a disome, head swiveling of the lead ribosome would force the colliding ribosome to accommodate this movement. Due to the interconnecting mRNA serving as a pivot, this most likely results in a overall movement of the colliding ribosome in the direction of the 60S of the lead ribosome. This probably further destabilizes the lead ribosome and contributes to efficient dissociation of the 60S subunit.
