# Supplementary material for: Structural basis for clearing of ribosome collisions by the RQT complex
Source: Nat Commun. 2023 Feb 17;14:921. doi: 10.1038/s41467-023-36230-8 (PMC9938168; doi:10.1038/s41467-023-36230-8)
Supplement: Supplementary file 5 — Reporting Summary [file 41467_2023_36230_MOESM5_ESM.pdf]

nature portfolio

Corresponding author(s): Roland Beckmann, Toshifumi Inada

Last updated by author(s): Jan 09, 2023

Reporting Summary

Nature Portfolio wishes to improve the reproducibility of the work that we publish. This form provides structure for consistency and transparency in reporting. For further information on Nature Portfolio policies, see our Editorial Policies and the Editorial Policy Checklist.

Statistics

For all statistical analyses, confirm that the following items are present in the figure legend, table legend, main text, or Methods section.

n/a	Confirmed	
☐	☒	The exact sample size (*n*) for each experimental group/condition, given as a discrete number and unit of measurement
☒	☐	A statement on whether measurements were taken from distinct samples or whether the same sample was measured repeatedly
☒	☐	The statistical test(s) used AND whether they are one- or two-sided<br>*Only common tests should be described solely by name; describe more complex techniques in the Methods section.*
☒	☐	A description of all covariates tested
☒	☐	A description of any assumptions or corrections, such as tests of normality and adjustment for multiple comparisons
☒	☐	A full description of the statistical parameters including central tendency (e.g. means) or other basic estimates (e.g. regression coefficient) AND variation (e.g. standard deviation) or associated estimates of uncertainty (e.g. confidence intervals)
☒	☐	For null hypothesis testing, the test statistic (e.g. *F*, *t*, *r*) with confidence intervals, effect sizes, degrees of freedom and *P* value noted<br>*Give P values as exact values whenever suitable.*
☒	☐	For Bayesian analysis, information on the choice of priors and Markov chain Monte Carlo settings
☒	☐	For hierarchical and complex designs, identification of the appropriate level for tests and full reporting of outcomes
☒	☐	Estimates of effect sizes (e.g. Cohen's *d*, Pearson's *r*), indicating how they were calculated

Our web collection on statistics for biologists contains articles on many of the points above.

Software and code

Policy information about availability of computer code

Data collection	EPU 2.12.1
Data analysis	Cryo-EM data were processed using Relion 3.1.1, MotionCor2 1.4.0, Gctf 1.06, CTFFIND4 4.1.13 and cryoSPARC 3.3.1. Molecular models were built and refined using WinCoot 0.9.6 and Phenix 1.19. Structural figures were created using ChimeraX 1.3.

For manuscripts utilizing custom algorithms or software that are central to the research but not yet described in published literature, software must be made available to editors and reviewers. We strongly encourage code deposition in a community repository (e.g. GitHub). See the Nature Portfolio guidelines for submitting code & software for further information.

Data

Policy information about availability of data

All manuscripts must include a data availability statement. This statement should provide the following information, where applicable:

- Accession codes, unique identifiers, or web links for publicly available datasets
- A description of any restrictions on data availability
- For clinical datasets or third party data, please ensure that the statement adheres to our policy

The cryo-EM structural data generated in this study have been deposited in the Protein Data Bank and the Electron Microscopy Data Bank under accession codes EMD-14861[https://www.ebi.ac.uk/emdb/search/EMD-14861] and PDB-7ZPQ [https://www.rcsb.org/structure/unreleased/7ZPQ] for the RQT-80S in C1 conformation; EMD-15280 [https://www.ebi.ac.uk/emdb/search/EMD-15280] for isolated, local refined RQT in 1 cnformation, EMD-15228 [https://www.ebi.ac.uk/

emdb/search/EMD-15228%20#] for 80S in C1 conformation; EMD-14921 and PDB-7ZRS for the RQT-80S in C2 conformation; EMD-14926 [https://www.ebi.ac.uk/emdb/search/EMD-14921] and PDB-7ZS5 [https://www.rcsb.org/structure/unreleased/7ZS5] for the 60S-peptidyl-tRNA complex; EMD-14978 [https://www.ebi.ac.uk/emdb/search/EMD-14978%20#] and PDB-7ZUW [https://www.rcsb.org/structure/unreleased/7ZUW] for the lead ribosome in the RQT-disome complex in C1 conformation; EMD-14979 [https://www.ebi.ac.uk/emdb/search/EMD-14979] and PDB-7ZUX [https://www.rcsb.org/structure/unreleased/7ZUX] for the collided RQT-80S ribosome in the RQT-disome complex.

Human research participants

Policy information about studies involving human research participants and Sex and Gender in Research.

Reporting on sex and gender	N/A
Population characteristics	N/A
Recruitment	N/A
Ethics oversight	N/A

Note that full information on the approval of the study protocol must also be provided in the manuscript.

Field-specific reporting

Please select the one below that is the best fit for your research. If you are not sure, read the appropriate sections before making your selection.

☒ Life sciences ☐ Behavioural & social sciences ☐ Ecological, evolutionary & environmental sciences

For a reference copy of the document with all sections, see nature.com/documents/nr-reporting-summary-flat.pdf

Life sciences study design

All studies must disclose on these points even when the disclosure is negative.

Sample size	All experiments were repeated at least three times independently. We described this in the figure legends.
Data exclusions	No data were excluded intentionally.
Replication	For each series of experiments, all replication attempts were successful.
Randomization	Randomization is not applicable for the experiments performed here.
Blinding	The investigators were not blinded during data collection.

Reporting for specific materials, systems and methods

We require information from authors about some types of materials, experimental systems and methods used in many studies. Here, indicate whether each material, system or method listed is relevant to your study. If you are not sure if a list item applies to your research, read the appropriate section before selecting a response.

Materials & experimental systems

n/a	Involved in the study	
☐	☒	Antibodies
☒	☐	Eukaryotic cell lines
☒	☐	Palaeontology and archaeology
☒	☐	Animals and other organisms
☒	☐	Clinical data
☒	☐	Dual use research of concern

Methods

n/a	Involved in the study	
☒	☐	ChIP-seq
☒	☐	Flow cytometry
☒	☐	MRI-based neuroimaging

Antibodies

Antibodies used	Anti-GFP antibody (Santa Cruz Biotechnology, Cat# sc-9996, clone B-2; 1:5000)<br>Anti-Flag antibody (Sigma-Aldrich, Cat# F1804, clone M2; 1:5000)<br>Anti-mouse HRP-Linked Whole Ab Sheep, horseradish Peroxidase (Cytiva Cat# NA931 ECL; 1:5000)

Anti-HA-Peroxidase High Affinity (3F10; Roche Cat# 12013819001, lot 34071100, 1:5000)
Anti-dioxygenin-AP (Roche Cat# 11093274910, 1:10000)

Validation

We used following antibodies.

For Western blottng in complementation and overexpression assays (Fig. 1e and 1f) Anti-GFP (Santa Cruz Biotechnology, Cat# sc-9996, clone B-2; 1:5000) and Anti-mouse HRP-Linked Whole Ab Sheep, horseradish Peroxidase (Cytiva Cat# NA931 ECL; 1:5000) were used. For the overexpression assay (Fig. 1f) Anti-Flag (Sigma-Aldrich, Cat# F1804, clone M2; 1:5000) was used. To check for ubiqitination of HA-tagged uS10 (Supplementary Fig. 1e and 1j) Anti-HA-Peroxidase High Affinity (3F10; Roche Cat# 12013819001, lot 34071100, 1:5000) was used. For Nortern blotting (Supplementary Fig. 1g) Anti-dioxygenin-AP (Roche Cat# 11093274910, 1:10000) was used to detect a DIG-labeled (CGN)12 mRNA probe.

All primary antibodies used in this study are commercial and have been validated by the manufacturers for use in S. cerevisiae.
